# Frequent daytime naps predict vocabulary growth in early childhood

**DOI:** 10.1111/jcpp.12583

**Published:** 2016-06-20

**Authors:** Klára Horváth, Kim Plunkett

**Affiliations:** ^1^ Department of Experimental PsychologyUniversity of OxfordOxfordUK

**Keywords:** Sleep, nap, children, infants, vocabulary development

## Abstract

**Background:**

The facilitating role of sleep for language learning is well‐attested in adults and to a lesser extent in infants and toddlers. However, the longitudinal relationship between sleep patterns and early vocabulary development is not well understood.

**Methods:**

This study investigates how measures of sleep are related to the development of vocabulary size in infants and toddlers. Day and night‐time sleeping patterns of infants and toddlers were compared with their concurrent and subsequent vocabulary development. Sleep assessments were conducted using a sleep diary specifically designed to facilitate accurate parental report. Sleep measures were used as predictors in a multilevel growth curve analysis of vocabulary development.

**Results:**

The number of daytime naps was positively associated with both predicted expressive (*p* = .062) and receptive vocabulary growth (*p* = .006), whereas the length of night‐time sleep was negatively associated with rate of predicted expressive vocabulary growth (*p* = .045). Sleep efficiency was also positively associated with both predicted receptive (*p* = .001) and expressive vocabulary growth (*p* = .068).

**Conclusions:**

These results point to a longitudinal relationship between sleep and language development, with a particular emphasis on the importance of napping at this age.

## Introduction

The importance of sleep for cognitive development in infancy has come under close scrutiny in recent years. Some studies have focused specifically on the impact of sleep on language development (Friedrich, Wilhelm, Born, & Friederici, 2015; Gomez, Bootzin, & Nadel, 2006; Horvath, Myers, Foster, & Plunkett, 2015), while others have focused on other aspects of the development of memory (Seehagen, Konrad, Herbert, & Schneider, 2015), or how early measures of sleep are associated with later cognitive outcome (Ednick et al., 2009). In this article, we ask whether there is a longitudinal relationship between infant vocabulary development and previously established patterns of sleep. Investigating links between sleep patterns and lexical development in infancy has the potential to contribute to our understanding of the manner in which sleep supports early language development and may allow us to identify groups that are at risk and provide early intervention.

The facilitating role of daytime naps on memory consolidation, generalisation and word learning in infancy has been confirmed by several studies. To investigate the cross‐sectional relationship between these cognitive processes and naps in infancy, researchers use study designs where they compare the performance of a nap group and a wake group. Seehagen et al. (2015) demonstrated the beneficial effect of a nap on declarative memories assessed with the deferred imitation task in 6‐ and 12‐month‐old infants. Naps also facilitated the abstraction of a rule in a language learning context in 15‐month olds (Gomez et al., 2006; Hupbach, Gomez, Bootzin, & Nadel, 2009), although this was not observed for 2.5‐year olds (Werchan & Gomez, 2014). Sleep has been shown to have a facilitating role on word learning and generalisation of word meanings in 9–16‐month olds in an event‐related potential study (Friedrich et al., 2015) and eye‐tracking studies provide behavioural evidence that naps facilitate both word learning (Horvath et al., 2015) and word generalisation in 16‐month‐old toddlers (Horvath, Liu, & Plunkett, 2016).

The evidence suggests that sleep measures can also predict later cognitive outcomes. Ednick et al. (2009) reviewed 13 studies investigating the longitudinal relationship between mental development and night‐time sleep in the first year of life. The reviewed studies used different methods for sleep assessment and mental assessment and they were carried out in infants at different ages. Moreover, some of them also included premature infants or infants with developmental delay. All of these factors may have contributed to the difficulty in drawing definitive conclusions. However, most of the studies found a predictive relationship between some kind of sleep measure (e.g. number of arousals, longest sleep period, sleep efficiency, REM storms, total sleep, total night‐time sleep etc.) and mental development. These associations were not exclusive to the first year of life. Short sleep duration during the night in toddlerhood was linked to poorer cognitive performance at school entry (Touchette et al., 2007), while circadian sleep regulation (i.e. the extent to which the sleep‐wake cycle is explained by a 24‐hr rhythm) at 19‐months was positively associated with cognitive performance (Dearing, McCartney, Marshall, & Warner, 2001). Furthermore, in an actigraphy study (Lam, Mahone, Mason, & Scharf, 2011) 3–5‐year‐old preschoolers with longer night‐time sleep had less impulsive errors in a neuropsychological task. On the other hand, Lam et al. reported a negative association between daytime naps and number recall. Of course, it is not possible to conclude from these studies whether the correlation between sleep and cognitive outcome reflects a true causal relationship or an overall maturational status which affects both (Ednick et al., 2009).

The relationship between infant sleep and vocabulary development has received less scrutiny. Circadian sleep regulation in 7‐ and 19‐month olds was positively correlated with language development at 36 months (Dearing et al., 2001) and a greater proportion of night‐time sleep to daytime sleep at 12 months correlated positively with a larger productive vocabulary at 26 months (Bernier, Carlson, Bordeleau, & Carrier, 2010). Similarly, sleep‐wake consolidation, defined as the proportion of daytime sleep and night‐time sleeping in a 24‐hr period at 6 and 18 months of age, was negatively correlated with later vocabulary scores. Moreover, children with language delay at 5 years had poorer sleep‐wake consolidation at 6 and 18 months. Genetic analyses of mono‐ and dizygotic twins showed that while sleep‐wake consolidation was highly heritable at 6 months, in 18‐month olds it was mainly due to shared environmental factors. Furthermore, the association between sleep and language showed a similar pattern, being genetically influenced at 6 months with a more significant role of the environment in older children (Dionne et al., 2011). Similar results were found for preschoolers. Lam et al. (2011) found a negative relationship between the duration of daytime naps and vocabulary size in preschoolers (3–5‐year olds), whereas vocabulary and night‐time sleep duration correlated positively. Sleep efficiency defined as the ratio between actual sleep time and the time spent in bed, has also been related to language development in atypically developing children (Edgin et al., 2015). These results suggest that night‐time sleep and daytime naps are of differing importance in vocabulary consolidation, and their relative influence might change with development. In the present study, our aim was to investigate further the relationship between vocabulary development and sleep in infants and toddlers, with a focus on individual differences as opposed to maturational changes in sleep patterns as in Dionne et al. (2011). Using growth curve modelling, we studied how patterns of sleep at different times of the day influence the growth of individual vocabulary sizes both in terms of the initial starting point and the pace of development. In particular, we asked whether daytime napping habits and night‐time sleep play the same facilitating role for infant vocabulary development. On the basis of experimental results showing the beneficial effects of daytime napping to vocabulary learning in toddlers (Horvath et al., 2015, 2016), we expected a positive relationship between daytime napping and later vocabulary size. Even though Lam et al. report evidence to the contrary, in the substantially younger age group of which our study consisted, we expected a greater importance of daytime sleep. Furthermore, as previous research positively related night‐time sleep and sleep efficiency with later cognitive functioning (Ednick et al., 2009), the beneficial role of night‐time sleep duration was hypothesised in vocabulary development.

## Methods

Families were recruited via phone, e‐mail or in person when they visited the laboratory for another experiment. Criteria for an infant's inclusion in the study were that parents did not indicate knowledge of any developmental problems and infants were full term (>37 weeks). As part of an initial assessment, parents were asked to fill in the Sleep and Naps Oxford Research Inventory (SNORI) and the Oxford Communicative Development Inventory (OCDI; Hamilton, Plunkett, & Schafer, 2000). The questionnaires, information about the study and a consent form were sent by post or delivered in person and returned by post. Follow‐up vocabulary assessments were scheduled 3 and 6 months after the initial assessment, again using the OCDI. Multiple deviations from the intended follow‐up resulted in variability in the timing and number of vocabulary assessments for each infant. However, all the infants reported in the study had an initial sleep and vocabulary assessment between 7.73 and 37.83 months of age. Follow‐up assessments of vocabulary varied between 2 and 8 occasions (median = 3), as some of the children participated in other studies in our laboratory in which the OCDI was taken. Participants without follow‐up data were also included as their data contributed to the initial assessments.

The study was approved by University of Oxford Central University Research Ethics Committee (MSD/IDREC/C2/2012/11).

### Questionnaires

#### Sleep and Naps Oxford Research Inventory

The SNORI is a sleep diary designed to be completed over 10 days. Ten days were chosen to include both weekends and weekdays even if a few days of data have to be excluded. Moreover, the likelihood of collecting at least 5 days of continuous data, permitting circadian analyses, is greatly enhanced. Additional questions about breastfeeding, travel between time zones, milestones of the infant's motor development and a survey about the most common health‐related factors, including current medication, which may affect the child's sleep, are also included in the inventory. The SNORI is an easily usable, handy sleep diary in the form of an A/5 booklet, which fits easily into handbags and onto bedside tables. The diary contains a separate page for each day with visual aids to distinguish daytime and night‐time. Parents are requested to draw a line to a time scale, which allows for a 15‐min accuracy, to indicate when their child had a nap or slept. Parents were instructed to indicate the time when they put their child to bed with a down arrow, and to fill in the time when their child was sleeping with a continuous line. If they woke up their child, they were asked to indicate it with an up arrow. If their children woke up on their own, they did not have to draw anything else. They were also asked to write down, under the line, the place and circumstances of the sleep or nap (e.g. bedroom, pushchair, car) and to take notes on night awakenings and nursery days. They were specifically asked every day whether there was anything unusual that day which might have disrupted their children's sleep. There is ample space for corrections and additional information which the parent may want to note. The colourful design of the SNORI helps to make it even more family friendly (Appendix S1). Parents were generally pleased with the format of the SNORI. Many of them gave unsolicited positive feedback as they preferred this format (i.e. drawing a line to a time scale) compared to a sleep diary in which they were asked to note down the specific sleep and wake‐up times. This way, they were also able to see for themselves any regularities in their child's sleep patterns. The SNORI is also available to view online and download from the Oxford BabyLab website (http://www.psy.ox.ac.uk/research/oxford-babylab/research-overview/sleep/snoriv3-1.pdf) or from the Appendix S1 of this article.^1^

#### Oxford Communicative Development Inventory

The OCDI (Hamilton et al., 2000) is a word list consisting of 416 words which are most commonly acquired in infancy. Parents are asked to indicate whether their child understands or understands and says a particular word. OCDI comprehension (i.e. receptive vocabulary) and OCDI production (i.e. expressive vocabulary) scores are derived by counting the number of words the child is assumed to understand or say. While the estimation of vocabulary production is fairly straightforward, it might be argued that parents are less precise in estimating their children's understanding of words. However, it has been shown that parents are quite accurate in assessing comprehension as well (Styles & Plunkett, 2009).

### Data processing

**Figure 1:**
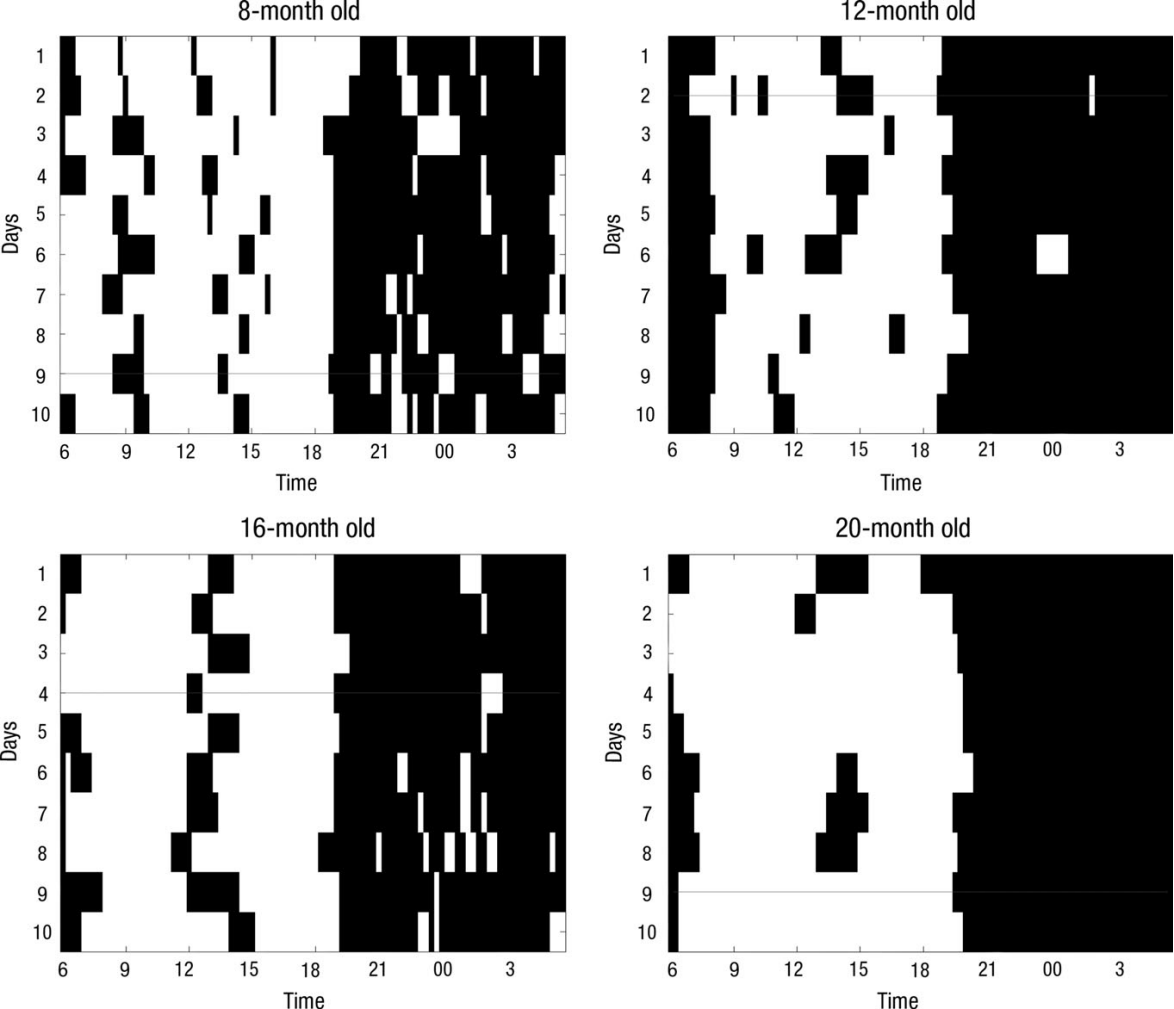
Sleep patterns of four children. Periods spent asleep are marked in black. The line indicates that the parent considered that day as special

The SNORI data were manually binary coded into Excel (Microsoft Office, 2013, Redmond, WA) and processed using MATLAB (version 8.2.0.701, R2013b; The MathWorks, Inc., Natick, MA). Any data returns for days which parents considered ‘special’ (e.g. due to illness or travelling) were excluded. Only those children were included in the analysis for whom we had at least 7 days of data, who did not take any medications and did not travel between time zones in the previous month of the study. Thirteen children were excluded from analysis due to incompleteness of their data provided. A diagram (Figure 1) of sleep patterns was created for each infant, which was checked manually to remove any days with peculiarities. Such presentation of the data helps researchers to obtain a picture of intra‐ and interindividual variability.

Onset of the main night‐time sleep was automatically derived for each day on the basis of a combination of variables including time of day and length of breaks in sleep using custom MATLAB routines. The algorithm used is as follows. First, we identified all the sleep starts and sleep ends within the data. Next, we defined a window between 6 p.m. and 8 a.m., as this is the time window which most probably contains the main sleep (i.e. longest sleep period which is normally during the night in our target age groups). We identified the first sleep start and the last sleep end within this period which were preliminarily assigned to the start and the end of the main sleep irrespective of the time spent awake between them. The algorithm then checked whether another sleep end preceded the main sleep start within the 4 p.m. to 6 p.m. period. If yes, and the difference was <1.5 hr between the main sleep start and the preceding sleep end, the corresponding sleep start replaced the main sleep start. Similarly, the algorithm checked whether another sleep start followed the main sleep end within the 8 a.m. to 10 a.m. period. If yes, and the difference was <1.5 hr between the main sleep end and the following sleep start, the corresponding sleep end replaced the main sleep end. If the difference was more than 1.5 hr in any of the cases, they were considered as daytime naps. The following sleep variables were derived for each day and were averaged within infants.

1. Sleep duration: length of the main sleep
2. Time spent awake during night
3. Total sleep time: sleep duration – time spent awake during night
4. Nap time: total duration of sleep other than the main night‐time sleep
5. Number of naps
6. Sleep efficiency: total sleep time/sleep duration

Statistical analyses were conducted in R (version 3.1.0; R‐Team, 2008).

### Statistical analyses

To analyse the possible effects of different predictors on the growth of receptive and expressive vocabulary, we used multilevel growth modelling following the procedures as described in Singer and Willett (2003). This approach takes into account both individual change (level‐1 submodel) and interindividual variability in this change (level‐2 submodel). Growth curve modelling has the additional advantage of accommodating longitudinal data which is unbalanced and where the intervals between data collection points differ. We used generalised linear mixed effect modelling with the *glmer* function of the *lme4* package for R (Bates, Maechler, Bolker, & Walker, 2015). Outcome measures were the OCDI comprehension and production scores which are binomially distributed (i.e. how many words the infant understands or understands *and* says out of 416 where the minimum is 0 when the infant did not know any words). As time spent awake during the night and sleep efficiency was highly correlated (*r*(246) = −.95, *p* < .001), we excluded time spent awake during the night from our analyses. We centred the age for the OCDI outcomes to 12 months. Total sleep time, nap time, number of naps and sleep efficiency were standardised, thereby reducing correlations between sleep variables to an acceptable level (|*r*| < .56). We provide results for the unconditional means models, the unconditional growth models and the full models with the final predictors selected using backward elimination. Predictors were removed stepwise and goodness‐of‐fit was compared across the models using the *anova* function. If model fit significantly worsened (as assessed comparing the log likelihoods), we reinstated the predictor even if it was not significant. The predictors used for vocabulary outcomes are listed in Table 1 with accompanying glosses. We only tested main effects for the initial status and the rate of change. Testing for interactions resulted in nonconvergence of the model due to the sample size.

**Table 1:** Predictors used for growth curve modelling of vocabulary outcome

Variable	Explanation
Age at OCDI	Age at vocabulary assessment
Total sleep time	Average time slept during night (time spent awake was excluded)
Nap time	Average time slept during day
Number of naps	Average number of naps
Sleep efficiency	The proportion of total sleep time and time spent asleep during night
Sex	
Breastfeeding	Was the infant breastfed at the time of the sleep assessment?

OCDI, Oxford Communicative Development Inventory.

## Results

### Participants

The data of 246 children (male: 121, female: 125) were analysed. Children were between 7.73‐ and 37.83‐months old at the initial assessment (mean = 17.89, *SD* = 6.34). Fifty‐seven of them were breastfed and 159 attended nursery at the initial assessment. The vast majority of mothers had at least an undergraduate degree (222). The average nap time was 114.67 (*SD* = 32.37) min, number of naps was 1.83 (*SD* = 0.93), total sleep time was 659.37 (*SD* = 51.61) min, time spent awake was 18.84 (*SD* = 26.13) min, sleep efficiency was 0.97 (*SD* = 0.04). At the initial OCDI assessment, the average receptive vocabulary size was 184.3 (*SD* = 139.08, range = 0–416), and expressive vocabulary score was 102.7 (*SD* = 137.27, range = 0–416). OCDI assessment took place at the ages 7.7–51.97 months.

### Sleep variables and age

To show how sleep variables change with age, we computed Pearson product moment correlation coefficients. Total sleep time (*r*(280) = .05, *p* = .4) and sleep duration (*r*(280) = −.03, *p* = .61) did not correlate significantly with age. Total awakening time (*r*(280) = −.17, *p* = .005), nap time (*r*(280) = −.53, *p* < .001), the number of naps (*r*(280) = −.49, *p* < .001) were negatively correlated with age, while sleep efficiency showed a positive association (*r*(280) = .2, *p* < .001).

### Cross‐sectional associations between sleep and vocabulary size

To investigate whether the sleep variables are associated with vocabulary size at the initial assessment independently of age, we performed a preliminary multiple regression with age and sleep variables included as potential predictors of vocabulary size. As expected, age was a highly significant predictor of expressive and receptive vocabulary scores (see Table 2). For comprehension, there was a significant positive association with sleep efficiency, indicating that infants who have less fragmented sleep tend to have larger receptive vocabularies. For production, sleep efficiency and the number of naps were also significant predictors and both were positively associated with expressive vocabulary size. Model parameters are shown in Table 2. The full model for comprehension explained 79.11% of the variance (*p* < .001), and for production, adjusted *R* ^2^ was .6891 (*p* < .001).

**Table 2:** Model parameters of the multiple regression analyses predicting vocabulary outcome from cross‐sectional variables

	Estimate	Standard error	*T*‐value	*p* value
Comprehension				
Intercept	−181.53	15.07	−12.05	<.001
Age	19.32	0.83	23.37	<.001
Nap time	−4.19	5.02	−0.84	.404
Sleep time	−7.95	4.93	−1.61	.108
Number of naps	9.69	7.61	1.27	.204
Sleep efficiency	15.47	7.85	1.97	.05
Production				
Intercept	−242.17	18	−13.45	<.001
Age	18.53	0.99	18.76	<.001
Nap time	−1.11	5.99	−0.19	.854
Sleep time	−2.58	5.88	−0.44	.662
Number of naps	19.19	9.01	2.11	.036
Sleep efficiency	19.99	9.37	2.13	.034

### Predictive relationships between sleep and vocabulary development

To explore how sleep and other control variables predict the initial status (reference: comprehension/production score at 12 months of age) and rate of receptive and expressive vocabulary development, growth curve modelling was used, allowing random effects for both initial status and rate of growth across children. After constructing the individual means and growth models, we stepped in all sleep variables as well as sex and breastfeeding. Insignificant predictors were removed stepwise provided this did not result in a poorer model fit. Model parameters for the unconditional mean, growth and final models are shown in Tables 3 and 4, for comprehension and production respectively. Model comparisons are shown in Tables 5 and 6.

**Table 3:** Mixed‐effect model parameters for OCDI comprehension score

	Unconditional means model				Unconditional growth model				Final model			
	Parameter	*SE*	*Z*	*p*	Parameter	*SE*	*Z*	*p*	Parameter	*SE*	*Z*	*p*
Fixed effects												
Initial status												
Intercept	−0.14	0.24	−0.60	.55	−2.49	0.1	−23.97	<.001	−2.5	0.11	−23.6	<.001
Sleep time									−0.1	0.07	−1.36	<.001
Sleep efficiency									−0.01	0.05	−0.2	.843
Breastfeeding (ref: formula fed)									0.08	0.06	1.19	.23
Rate of change												
Age					0.34	0.01	33.8	<.001	0.31	0.01	25.35	<.001
Number of naps									0.03	0.01	3.21	.001
Sleep efficiency									0.03	0.01	2.76	.006
Sex (ref: male)									0.05	0.01	3.78	<.001

	Variance	*SD*	Variance	*SD*	Variance	*SD*
Random effects						
Intercept	9.01	3	2.14	1.46	2.05	1.43
Age	0.15	0.39	0.02	0.14	0.02	0.13

OCDI, Oxford Communicative Development Inventory; *SE*, standard error; *SD*, standard deviation.

**Table 4:** Mixed‐effect model parameters for OCDI production score

	Unconditional means model				Unconditional growth model				Final model			
	Parameter	*SE*	*Z*	*p*	Parameter	*SE*	*Z*	*p*	Parameter	*SE*	*Z*	*p*
Fixed effects												
Initial status												
Intercept	−0.75	0.31	−2.4	.016	−5.6	0.18	−31.26	<.001	−5.58	0.19	−29.73	<.001
Nap time									−0.27	0.19	−1.39	.16
Rate of change												
Age					0.48	0.02	30.59	<.001	0.43	0.02	23.13	<.001
Nap time									0.02	0.02	1.25	.212
Number of naps									0.03	0.02	1.86	.062
Sleep time									−0.02	0.01	−2.01	.045
Sleep efficiency									0.03	0.02	1.83	.068
Breastfeeding (ref: formula fed)									0.01	0.01	1.55	.122
Sex (ref: male)									0.09	0.02	5.4	<.001

	Variance	*SD*	Variance	*SD*	Variance	*SD*
Random effects						
Intercept	34.45	5.87	6.84	2.62	6.92	2.63
Age	0.31	0.55	0.05	0.22	0.05	0.22

CI, 95% confidence interval; OCDI, Oxford Communicative Development Inventory; *SE*, standard error; *SD*, standard deviation.

**Table 5:** Model comparisons for OCDI comprehension

	*df*	AIC	BIC	logLik	Deviance	*χ* ^2^	*df*	*p*
Unconditional means model	4	8488.5	8506.5	−4240.3	8480.5			
Unconditional growth model	5	8067.4	8089.9	−4028.7	8057.4	423.11	1	<.001
Final model	11	7509.4	7558.1	−3743.7	7487.4	570.02	6	<.001

*df*, degrees of freedom; AIC, Akaike Information Criterion; BIC, Bayesian Information Criterion; logLik, log likelihood; OCDI, Oxford Communicative Development Inventory.

**Table 6:** Model comparisons for OCDI production

	*df*	AIC	BIC	logLik	Deviance	*χ* ^2^	*df*	*p*
Unconditional means model	4	7786.9	7804.9	−3889.5	7778.9			
Unconditional growth model	5	7,386	7408.5	−3,688	7,376	402.95	1	<.001
Final model	12	6867.5	6920.6	−3421.7	6843.5	532.5	7	<.001

*df*, degrees of freedom; AIC, Akaike Information Criterion; BIC, Bayesian Information Criterion; logLik, log likelihood; OCDI, Oxford Communicative Development Inventory.

For comprehension, the number of naps and sleep efficiency along with sex had a significant effect on the predicted rate of development. Children who took more daytime naps had a higher rate of predicted receptive vocabulary growth, as did girls in our sample. Furthermore, those children who had more efficient sleep had a higher rate of predicted receptive vocabulary development. Although not significant in itself, the removal of the effect of breastfeeding and night‐time sleep on the initial status resulted in a poorer model fit. Therefore, breastfeeding and night‐time sleep duration were included in the final model.

**Figure 2:**
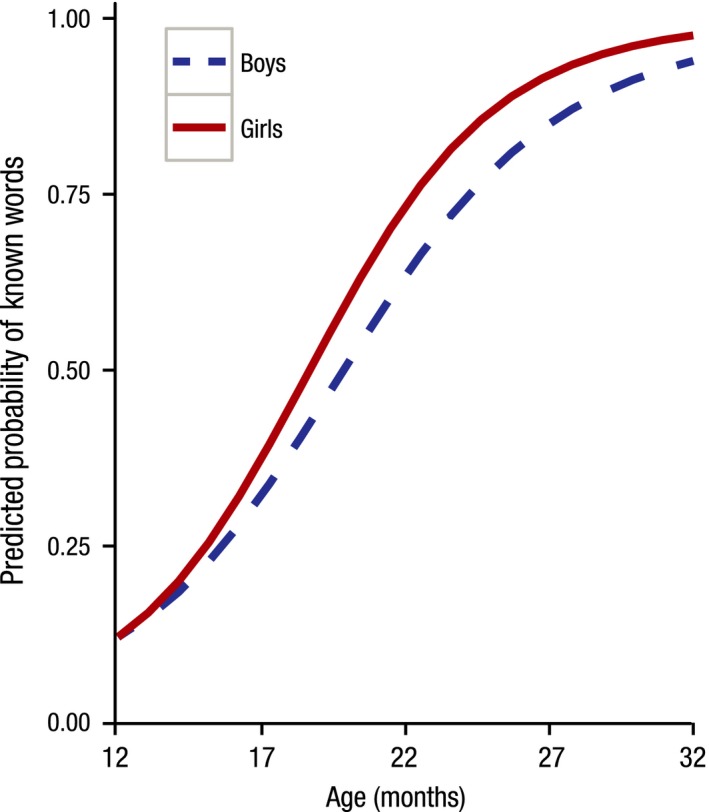
Receptive vocabulary development by sex. Girls have a faster rate of vocabulary growth

**Figure 3:**
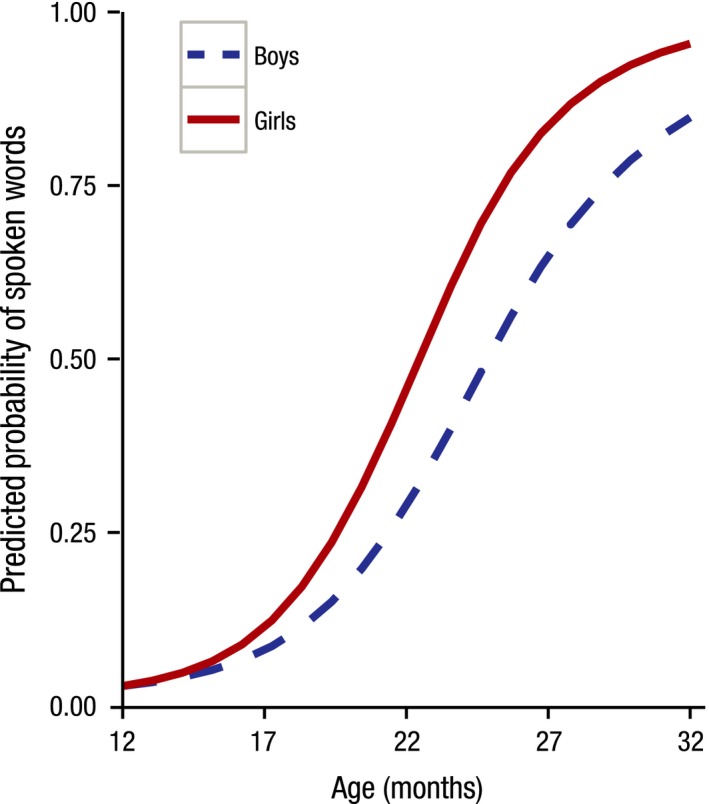
Expressive vocabulary development by sex. Girls have a faster rate of vocabulary growth

For predicted production, the effect of sleep time and sex had a significant impact on the rate of vocabulary growth: longer night‐time sleep resulted in decreased predicted growth of expressive vocabularies. The effect of sleep efficiency and the number of naps approached significance: children who had more naps during the day and had better sleep efficiency tended to have enhanced predicted growth. Although not significant, the effects of nap time on initial status and the predicted rate of vocabulary growth and the effects of breastfeeding on vocabulary growth contributed to a significantly better model fit, and so were kept in the model. Somewhat unsurprisingly, both receptive and expressive vocabularies of girls developed at a faster pace than boys’ (Figures 2 and 3).

**Figure 4:**
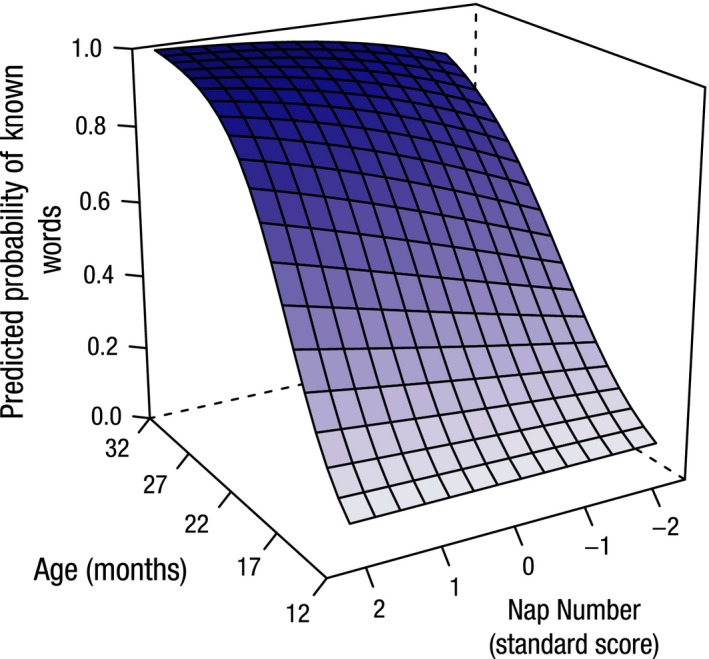
The effect of the number of naps on receptive vocabulary development with age. Standard scores are displayed for number of naps. Children with more daytime naps show a faster rate of receptive vocabulary development with age

**Figure 5:**
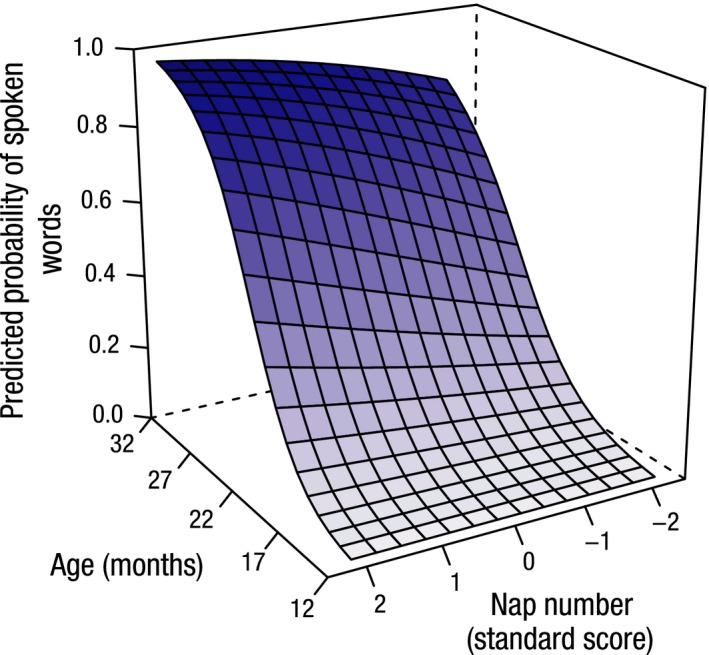
The effect of the number of naps on expressive vocabulary development with age. Standard scores are displayed for number of naps. Children with more daytime naps show a faster rate of expressive vocabulary development with age

**Figure 6:**
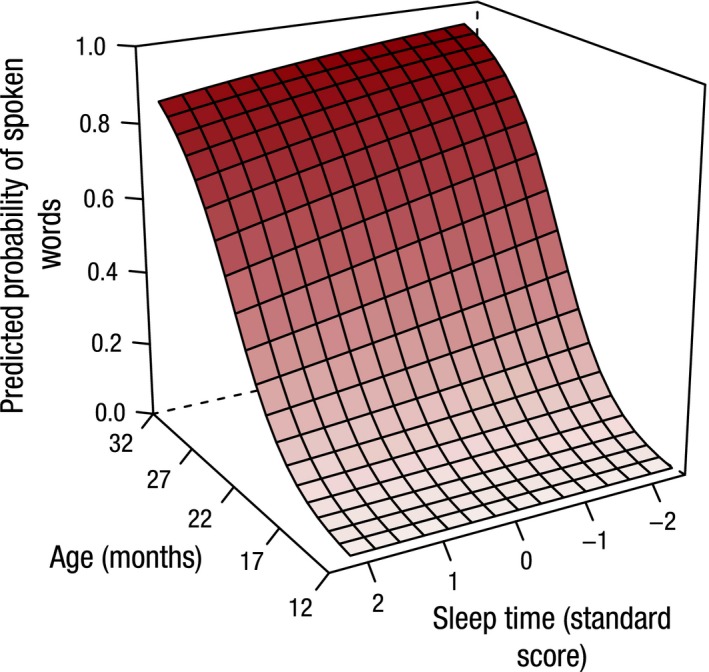
The effect of sleep time on expressive vocabulary development with age. Standard scores are displayed for sleep time. Children with longer night‐time sleep show a slower rate of expressive vocabulary development with age

Figure 4 depicts the model's representation of the relationship between receptive vocabulary size for age and the number of naps and highlights the finding that children with more daytime naps show more rapid predicted growth of receptive vocabulary. A similar relationship for predicted expressive vocabulary scores is depicted in Figure 5. Figure 6 highlights the finding that children with shorter night‐time sleep master all the words in production on the OCDI at younger ages.

## Discussion

Our aim in this study was to investigate how individual differences in daytime and night‐time sleep contribute to vocabulary development. After an initial sleep and vocabulary assessment, we assessed vocabulary development in infants and toddlers on a minimum of two and up to eight occasions. This design permitted us to model the vocabulary development of each child and investigate the extent to which the measured sleep variables predicted individual differences between children. We found that more frequent daytime napping was positively associated with the rate of both predicted receptive and expressive vocabulary development, although the latter was only a marginal effect. In addition, those who slept less during the night exhibited a larger rate of predicted expressive vocabulary growth. Furthermore, less fragmented sleep was associated with significantly larger predicted receptive vocabulary scores and marginally greater predicted expressive vocabularies. Perhaps unsurprisingly, the statistical growth curve analysis revealed that girls develop more quickly in terms of their vocabulary size. Overall, these findings point to a clear longitudinal relationship between early sleeping patterns and later vocabulary development.

The positive association between daytime sleep and vocabulary development may seem unsurprising in the light of recent studies showing that a daytime nap helps to consolidate new word forms (Friedrich et al., 2015; Horvath et al., 2015) and facilitates the generalisation of novel word meanings (Friedrich et al., 2015; Horvath et al., 2016). Furthermore, studies with 15‐month olds (Hupbach et al., 2009) and preschoolers (Kurdziel, Duclos, & Spencer, 2013) found that after a nap children retain the information they had learnt for at least 24 hr in contrast to children who did not have a nap after learning. One interpretation of this pattern of findings is that because infants’ memory systems, especially the hippocampal short‐term stores, have smaller capacity and are probably more prone to interference (for review see Mullally & Maguire, 2014), more frequent naps are needed to efficiently consolidate new information (Kurdziel et al., 2013). The fact that the frequency of napping was a stronger predictor than the duration of daytime sleep in the present study highlights the advantage of frequent distinct sleep periods for memory consolidation over a long nap during the day, consistent with the interpretation offered above. It is known that specific hippocampal structures and layers particularly in the CA3 region mature later (Lavenex & Banta Lavenex, 2013), which might underlie the differential need for sleep‐dependent memory consolidation in adults and young children. This interpretation seems to be further supported by the finding that the performance of nonhabitual nappers decreased less over wake than that of habitual nappers (Kurdziel et al., 2013). Interestingly, the age (3–5 years) when the hippocampus reaches its adult capacity (Lavenex & Banta Lavenex, 2013; Mullally & Maguire, 2014) co‐occurs with the age when sleep becomes monophasic in children. On the other hand, Gomez and Edgin (2015) argue that for infants younger than 18‐months old, sleep‐dependent memory consolidation cannot be the consequence of hippocampal replay during sleep due to the immaturity of hippocampal circuits, although it might reflect sleep‐dependent consolidation of cortical learning perhaps through synaptic descaling (Tononi & Cirelli, 2014). Irrespective of whether an immature hippocampus or neocortex is responsible for learning in infants and toddlers, it seems that more frequent sleep periods makes the consolidation processes more efficient. In contrast to daytime naps, the finding that night‐time sleep was negatively associated with predicted expressive vocabulary size may seem somewhat counterintuitive. However, similar results have been found in younger infants’ general cognitive development. In neonates, the longest sleep period was negatively associated with a later cognitive outcome (Anders, Keener, & Kraemer, 1985; Freudigman & Thoman, 1993). A similar relationship was found in premature infants at 36 weeks of gestational age (Gertner et al., 2002). Infants who slept more over 24 hr and slept more during the night (over a 12‐hr period) had a lower index of mental development at 6 months. These findings may be explained by supposing that shorter sleep duration is an index of a more mature sleep pattern (Anders et al., 1985; Gertner et al., 2002). Alternatively, the increased duration of the longest sleep period may reflect a higher stress reactivity, thus a greater vulnerability, which may be associated with less than optimal cognitive development (Freudigman & Thoman, 1993). Furthermore, it is also possible that those children who slept less during the night, had more opportunities for having more naps during the day, which may facilitate expressive vocabulary growth.

It is important to note that our results are not consistent with the two comparable studies in similar age groups. Dionne et al. (2011) found no relationship between night‐time sleep and vocabulary outcome at 18 and 30 months (although there was a positive correlation between night‐time sleep at 18 months and vocabulary at 60 months) and reported a negative association between daytime sleep at 6 months and later vocabulary score. However, there are considerable differences between Dionne et al.'s study and that reported here. Dionne et al.'s findings are based on Spearman correlations, and therefore, offer no control for confounding factors. Nevertheless, the authors provide information from structural equation modelling with the appropriate control for other variables, but only for the measure of sleep‐wake consolidation. Although it is not straightforward to compare, the negative association between sleep‐wake consolidation and vocabulary outcome still suggests a contrasting set of results to that of the present study. Our methodology provides more accurate measures of sleep, as we used sleep diaries for a minimum of 7 days permitting parents to indicate sleep times with a 15‐min accuracy. Dionne et al. (2011) used interviews with rounded hours as preset answers for questions making it difficult to investigate more subtle individual differences. Moreover, we defined the total night‐time sleep as the difference of the sleep duration and the time spent awake during night. One major advantage of Dionne et al.'s study is their sample size of 1,029, although it is important to note that the sample consisted of twins. Furthermore, they carried out the sleep assessment at fixed ages: 6, 18 and 30 months, whereas we studied children from a wider age range. In another comparable study, Lam et al. (2011) found that nap time correlated negatively with vocabulary scores, while sleep time correlated positively with vocabulary scores in 3–5‐year‐old preschoolers. It is important to highlight the substantially different age range of our participants indicating that there may be different relationships between sleep variables and vocabulary size at different stages of development. Moreover, Lam et al. (2011) used a cross‐sectional design, therefore, the correlations found may reflect mainly maturational changes. It is important to note that the number of naps during the day has not been related to vocabulary learning in previous studies which might account for some of the inconsistencies. On the basis of our results, the number of naps seems to be a more important predictor than the duration of daytime sleeping. Thus, future studies should include nap frequency in their analyses.

We also found sleep efficiency and sex as significant predictors of predicted vocabulary development. Sleep efficiency has been shown to be important for expressive vocabulary development in toddlers with Down syndrome (Edgin et al., 2015). In addition, more fragmented sleep was associated with a lower mental developmental index at 10 months of age (Scher, 2005). Our results are not only consistent with these findings but they also show that a longitudinal relationship exists between sleep efficiency and vocabulary development. Regarding the effect of sex, faster vocabulary development of girls is well‐attested in the literature (Mayor & Plunkett, 2011; Westerlund & Lagerberg, 2008). Thus, our results are convergent.

While our study describes the associations between sleep and vocabulary development in typically developing children, it has clear implications for clinical populations. First, it highlights that children with sleep disorders and disrupted daytime sleep patterns may be at higher risk for having less developed language skills. Second, it suggests that improving sleep in atypically developing children with disturbed sleeping (e.g. Down syndrome, Williams syndrome) may be beneficial for their language development. Third, it is possible that children whose cognitive impairment is of unknown origin may suffer from an underlying sleep disturbance. However, further studies are needed to investigate this relationship in greater depth for clinical populations, with particular reference to the question of how the frequency of napping influences learning.

One major limitation of our study is that our sample was homogeneous in terms of maternal education, placing a limit on how much our findings can be generalised. Furthermore, we conducted the sleep assessment at only one instance and the age of the assessment varied across children. Multilevel statistical modelling is able to handle this type of sampling provided sufficient data points are collected for each participant. Nevertheless, following both sleep and vocabulary development longitudinally could provide important further insights into their relationship. Similarly, because the majority of our samples were between the ages of 12–38 months, we think that the validity of our predictions are limited to this age range. Furthermore, even though sleep diaries have been shown to be fairly accurate (Kaplan, Talbot, Gruber, & Harvey, 2012; Werner, Molinari, Guyer, & Jenni, 2008), they cannot provide as accurate data as physiological measures. This is especially true for measuring the number and the time of awakenings since parents are often not aware of their children waking up during the night. Therefore, studies using actigraphy or polysomnography should be conducted to confirm our results. Furthermore, our models did not allow for interactions between sleep variables and age because of constraints due to sample size. Hence, it is impossible to derive whether every age group contributed similarly to the results found. Longitudinal studies with narrower age groups would be useful to further investigate this question. Moreover, although parental vocabulary questionnaires have shown to be reliable tools in assessing children's vocabulary scores, they may be less reliable at older ages as the children know more words. In addition, production scores may be more accurate since they are easier for parents to assess.

## Conclusions

Taken together, our results shed light on some of the associations between sleep and vocabulary development. Since inconsistencies emerged in the literature, future studies are warranted to disentangle this relationship. Understanding the connection better may allow us to identify the risk of impaired vocabulary development and intervene on time.

**Key points**

- Cross‐sectional studies have provided evidence that daytime napping enhances several cognitive processes in early childhood including word learning.
- Our study showed that a longitudinal relationship also exists between different sleep variables and vocabulary development.
- More frequent daytime napping predicted both larger receptive and expressive vocabulary scores.
- Night‐time sleeping was negatively associated with expressive vocabulary scores.
- Parents and child practitioners should be advised to promote daytime napping in early childhood.

## Supporting information


**Appendix S1.** Sleep and Naps Oxford Research Inventory (SNORI).

## Note

1 Researchers are welcome to use this diary and modify it to fit their needs providing this article is cited appropriately.
