# Supplementary material for: Frequent daytime naps predict vocabulary growth in early childhood
Source: J Child Psychol Psychiatry. 2016 Jun 20;57(9):1008–17. doi: 10.1111/jcpp.12583 (PMC5017299; doi:10.1111/jcpp.12583)
Supplement: Supplementary file 1 — Appendix S1. Sleep and Naps Oxford Research Inventory (SNORI). [file JCPP-57-1008-s001.pdf]

Oxford Centre for Developmental Science

BABY LAB Oxford University

Sleep and Naps
Oxford Research Inventory

UNIVERSITY OF OXFORD

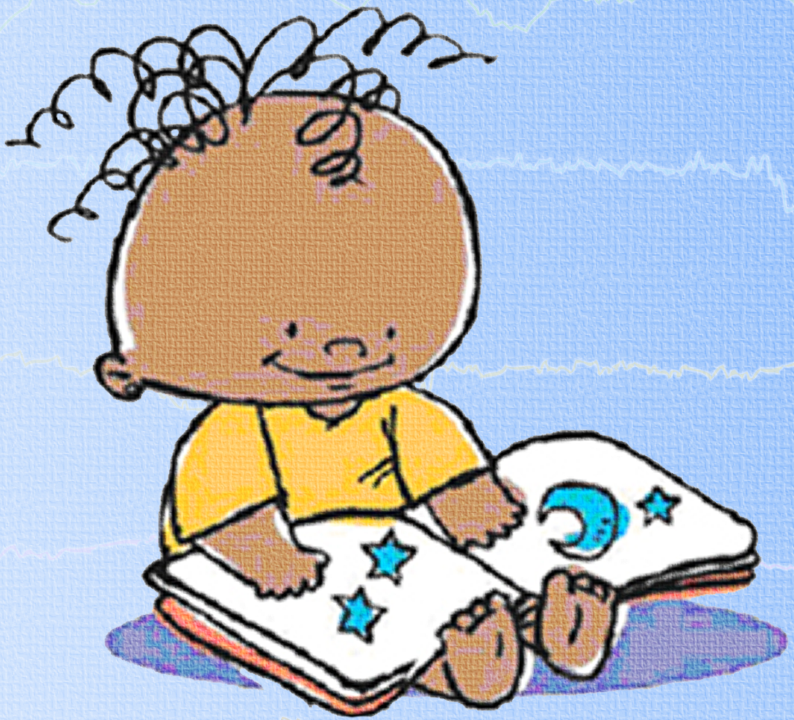

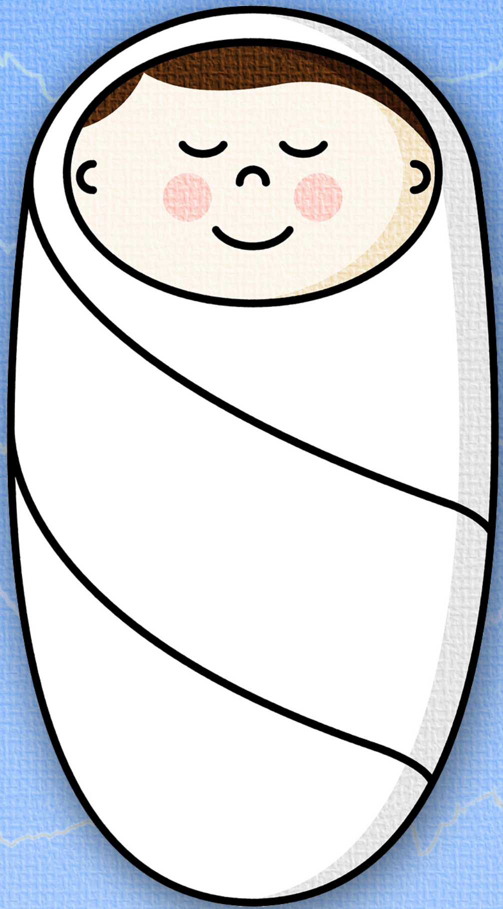

Introduction

Dear Parent,

Our infants spend more than half the day sleeping, and yet they acquire new skills and knowledge rapidly. We have good reason to believe that sleep is not just passive resting, but it plays an active role in brain development. Our goal is to study how sleep and naps change with time and to investigate the factors that may influence them. To get a picture of your child's sleep, we would like you to complete a sleep and nap diary for your child for ten days in a row. We are interested in your child's normal, daily sleep and how it varies from one day to the next. We also would like you to answer questions about general sleeping habits and development of your child.

We highly appreciate your help!

Thank you for your help!

If you have any questions feel free to contact me: horv.klara@gmail.com

DAY __ TODAY'S DATE:

Daytime 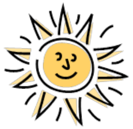 **Sleep & Naps**

6 AM 7 AM 8 AM 9 AM 10 AM 11 AM 12 PM

12 PM 1 PM 2 PM 3 PM 4 PM 5 PM 6 PM

Night time 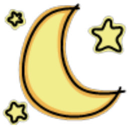 **Sleep & Naps**

6 PM 7 PM 8 PM 9 PM 10 PM 11 PM 12 AM

12 AM 1 AM 2 AM 3 AM 4 AM 5 AM 6 AM

Was there anything unusual (e.g. activity/illness) which might have disrupted your child's sleep today?

Your baby

Name of your child: ____________________

Date of birth (DD/MM/YYYY): ____________________

Due date (DD/MM/YYYY): ____________________

Sex: **F** **M**

Parents' names: ____________________

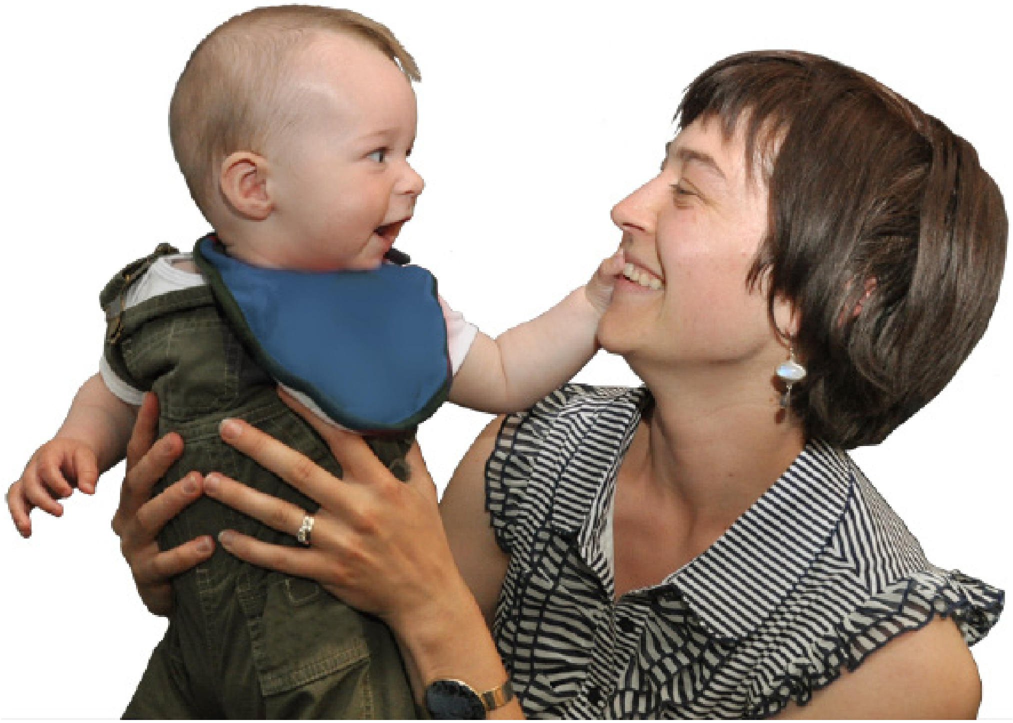

Motor Development

The following statements related to your child's motor development. Please indicate if they are true or false for your child, and if true, you can give how old was your baby when he/she has first started to do the specific skill.

My child...	True	Not yet	Age of ac-quisition (months)
rolls from front to back			
sits without support			
stands with assistance			
crawls on hands and knees			
walks with assistance			
stands alone			
walks alone			
climbs steps with support			
runs			
kicks ball			
reaches, pulls object to mouth			
uses thumb for grasping			
draws line			
scribbles			
undresses			
draws a circle			

Day ___

Today's date:

Daytime 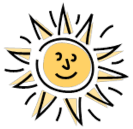 **Sleep & Naps**

6 AM 7 AM 8 AM 9 AM 10 AM 11 AM 12 PM

12 PM 1 PM 2 PM 3 PM 4 PM 5 PM 6 PM

Night time 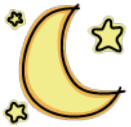 **Sleep & Naps**

6 PM 7 PM 8 PM 9 PM 10 PM 11 PM 12 AM

12 AM 1 AM 2 AM 3 AM 4 AM 5 AM 6 AM

Was there anything unusual (e.g. activity/illness) which might have disrupted your child's sleep today?

DAY 10 TODAY'S DATE:

Daytime 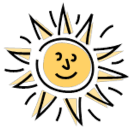 **Sleep & Naps**

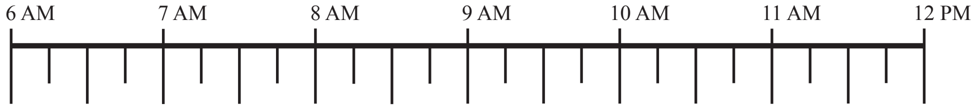

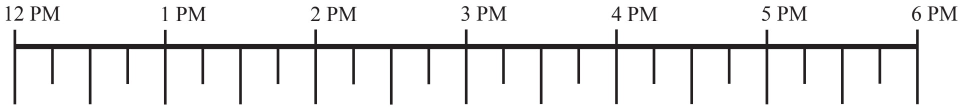

Night time 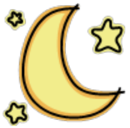 **Sleep & Naps**

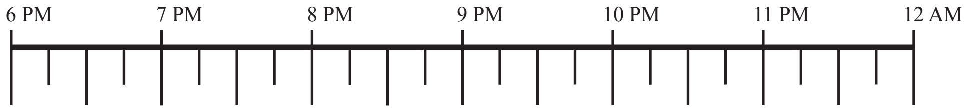

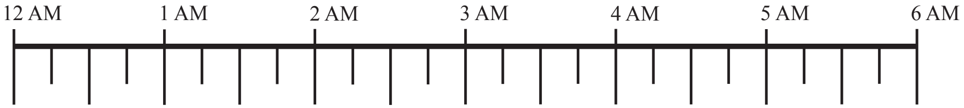

Was there anything unusual (e.g. activity/illness) which might have disrupted your child's sleep today?

HEALTH AND MEDICATION

Have you travelled with your child more than two timezones in the past month?

☐ Yes ☐ No

Does your child take any medication on a regular basis? If yes, please provide the name of the drug.

**My child takes medication for**	**No**	**Yes**	**Name of the drug**
his/her sleep (e.g. melatonin)			
epilepsy			
heart disease (e.g. antiar-rythmic drugs)			
allergy			
other			

Does your child have any vision or other eye problem?

☐ Yes ☐ No

Does your child suffer from allergies?

☐ Yes, allergic to ______________ ☐ No

Does your child suffer from asthma?

☐ Yes ☐ No

Does your child snore?

☐ Never ☐ Sometimes (less than 2 nights/week)

☐ Often (3-5 nights/week) ☐ Always (6-7 nights/week)

Does your child stop breathing during sleep?

☐ Never ☐ Sometimes (less than 2 nights/week)

☐ Often (3-5 nights/week) ☐ Always (6-7 nights/week)

SLEEP DIARY

Instructions

Please indicate with a down arrow (↓) the time when you put your child to bed and with a continuous line please fill in the time when your child was sleeping. If your baby woke up on his/her own, you do not need to draw anything else. If you woke your baby, you can indicate this with an up arrow (↑). Under the line please write down the place and circumstances of the sleep or nap (e.g. bedroom, pushchair, car). Please take notes on night awakenings and nursery days as well.

Below, you can see an example.

Examples

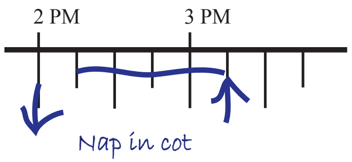

Jane put her daughter to bed for a nap at 2PM and she fell asleep 15 minutes later. She napped for 1 hour, when Jane woke her up to go to the shops.

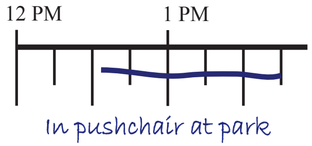

Baby George fell asleep on his own in his puschair at 12.30 PM and he slept until 1.45 PM when he woke up by himself.

DAY 9

TODAY'S DATE:

Daytime

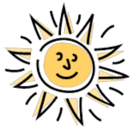

Sleep & Naps

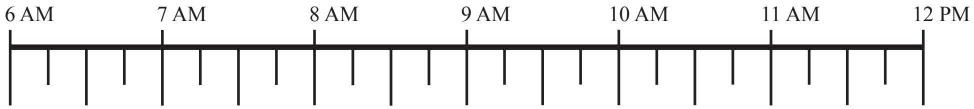

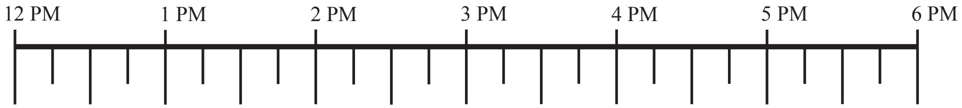

Night time

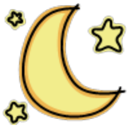

Sleep & Naps

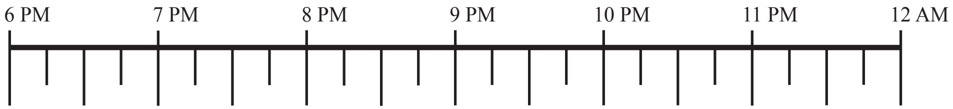

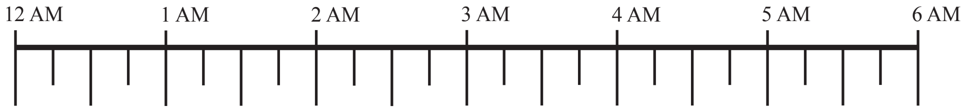

Was there anything unusual (e.g. activity/illness) which might have disrupted your child's sleep today?

DAY 8

TODAY'S DATE:

Daytime Sleep & Naps

6 AM 7 AM 8 AM 9 AM 10 AM 11 AM 12 PM

12 PM 1 PM 2 PM 3 PM 4 PM 5 PM 6 PM

Night time Sleep & Naps

6 PM 7 PM 8 PM 9 PM 10 PM 11 PM 12 AM

12 AM 1 AM 2 AM 3 AM 4 AM 5 AM 6 AM

Was there anything unusual (e.g. activity/illness) which might have disrupted your child's sleep today?

DAY 1

TODAY'S DATE:

Daytime Sleep & Naps

6 AM 7 AM 8 AM 9 AM 10 AM 11 AM 12 PM

12 PM 1 PM 2 PM 3 PM 4 PM 5 PM 6 PM

Night time Sleep & Naps

6 PM 7 PM 8 PM 9 PM 10 PM 11 PM 12 AM

12 AM 1 AM 2 AM 3 AM 4 AM 5 AM 6 AM

Was there anything unusual (e.g. activity/illness) which might have disrupted your child's sleep today?

DAY 2

TODAY'S DATE:

Daytime Sleep & Naps

6 AM | 7 AM | 8 AM | 9 AM | 10 AM | 11 AM | 12 PM

12 PM | 1 PM | 2 PM | 3 PM | 4 PM | 5 PM | 6 PM

Night time Sleep & Naps

6 PM | 7 PM | 8 PM | 9 PM | 10 PM | 11 PM | 12 AM

12 AM | 1 AM | 2 AM | 3 AM | 4 AM | 5 AM | 6 AM

Was there anything unusual (e.g. activity/illness) which might have disrupted your child's sleep today?

DAY 7

TODAY'S DATE:

Daytime Sleep & Naps

6 AM | 7 AM | 8 AM | 9 AM | 10 AM | 11 AM | 12 PM

12 PM | 1 PM | 2 PM | 3 PM | 4 PM | 5 PM | 6 PM

Night time Sleep & Naps

6 PM | 7 PM | 8 PM | 9 PM | 10 PM | 11 PM | 12 AM

12 AM | 1 AM | 2 AM | 3 AM | 4 AM | 5 AM | 6 AM

Was there anything unusual (e.g. activity/illness) which might have disrupted your child's sleep today?

DAY 6

TODAY'S DATE:

Daytime

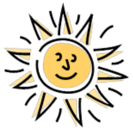

Sleep & Naps

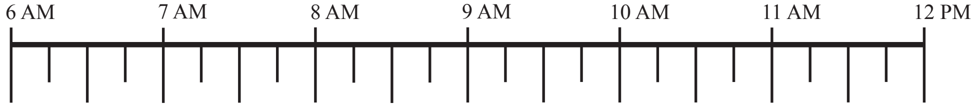

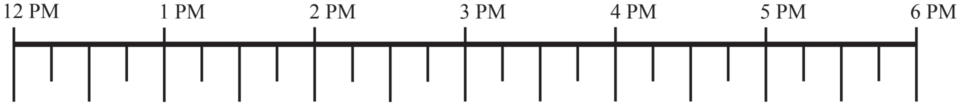

Night time

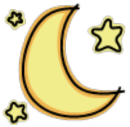

Sleep & Naps

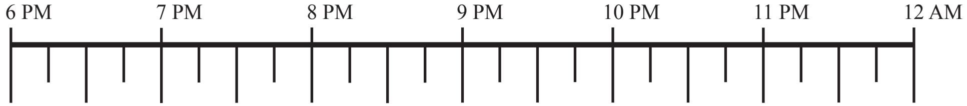

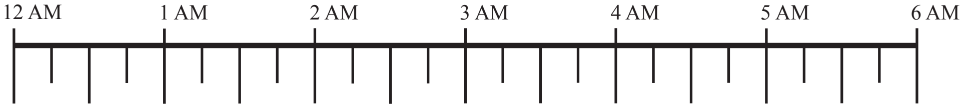

Was there anything unusual (e.g. activity/illness) which might have disrupted your child's sleep today?

DAY 3

TODAY'S DATE:

Daytime

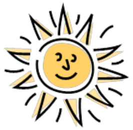

Sleep & Naps

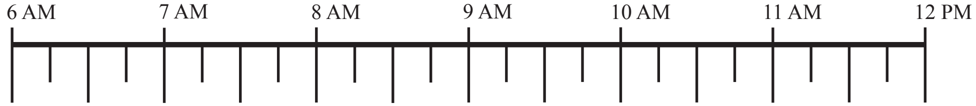

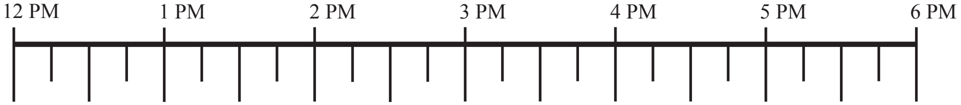

Night time

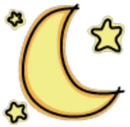

Sleep & Naps

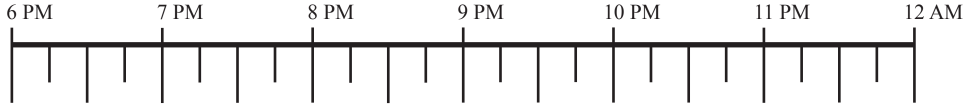

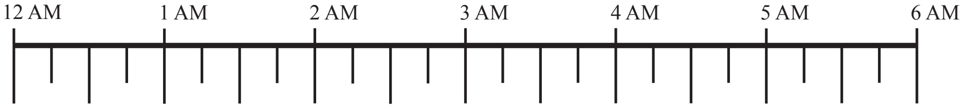

Was there anything unusual (e.g. activity/illness) which might have disrupted your child's sleep today?

DAY 4

TODAY'S DATE:

Daytime 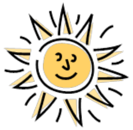 **Sleep & Naps**

6 AM 7 AM 8 AM 9 AM 10 AM 11 AM 12 PM

12 PM 1 PM 2 PM 3 PM 4 PM 5 PM 6 PM

Night time 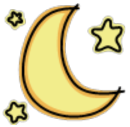 **Sleep & Naps**

6 PM 7 PM 8 PM 9 PM 10 PM 11 PM 12 AM

12 AM 1 AM 2 AM 3 AM 4 AM 5 AM 6 AM

Was there anything unusual (e.g. activity/illness) which might have disrupted your child's sleep today?

DAY 5

TODAY'S DATE:

Daytime 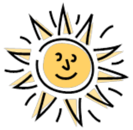 **Sleep & Naps**

6 AM 7 AM 8 AM 9 AM 10 AM 11 AM 12 PM

12 PM 1 PM 2 PM 3 PM 4 PM 5 PM 6 PM

Night time 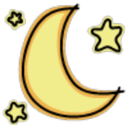 **Sleep & Naps**

6 PM 7 PM 8 PM 9 PM 10 PM 11 PM 12 AM

12 AM 1 AM 2 AM 3 AM 4 AM 5 AM 6 AM

Was there anything unusual (e.g. activity/illness) which might have disrupted your child's sleep today?
